# Phylogenetic analysis of the sharpshooter genus *Subrasaca* Young, 1977 (Hemiptera, Cicadellidae, Cicadellini)

**DOI:** 10.3897/zookeys.484.9264

**Published:** 2015-02-27

**Authors:** Roberta dos Santos da Silva, Gabriel Mejdalani, Rodney R. Cavichioli

**Affiliations:** 1 Departamento de Entomologia, Museu Nacional, Universidade Federal do Rio de Janeiro, Quinta da Boa Vista, São Cristóvão, 20940-040, Rio de Janeiro, RJ, Brasil; 2 Departamento de Zoologia, Setor de Ciências Biológicas, Universidade Federal do Paraná, Caixa Postal 19020, 81531-980, Curitiba, PR, Brasil; 3 Pós-graduação em Zoologia, Museu Nacional, Universidade Federal do Rio de Janeiro

**Keywords:** Auchenorrhyncha, Cicadellinae, cladistics, Membracoidea, phylogeny

## Abstract

The South American sharpshooter genus *Subrasaca* comprises 14 species. Some species of this genus are quite common in the Brazilian Atlantic Rainforest. In this paper, a phylogenetic analysis of *Subrasaca*, based on a matrix of 20 terminal taxa and 72 morphological characters of the head, thorax, and male and female genitalia, is presented. The analysis yielded six equally most parsimonious trees (197 steps, CI = 0.6091, RI = 0.5722, and RC = 0.3486). The results suggest that *Subrasaca* is a monophyletic taxon, although the genus branch is not robust. The clade showing the highest bootstrap and Bremer scores is formed by species with longitudinal dark brown to black stripes on the forewings (*Subrasaca bimaculata*, *Subrasaca constricta*, *Subrasaca curvovittata*, and *Subrasaca flavolineata*), followed by *Subrasaca atronasa* + *Subrasaca austera*.

## Introduction

The infraorder Cicadomorpha comprises three superfamilies, Cicadoidea (cicadas), Cercopoidea (spittlebugs or froghoppers), and Membracoidea (leafhoppers and treehoppers). According to Hamilton (1999), the monophyly of the Cicadomorpha is well-supported by morphological synapomorphies, including the presence of a complex filter chamber. Cryan (2005), based on molecular data (18S rDNA, 28S rDNA, and histone 3), also supports the monophyly of the Cicadomorpha and suggests the following relationships for the superfamilies: (Membracoidea (Cicadoidea, Cercopoidea)). Based both on morphological and molecular data, the monophyly of the Membracoidea is also well-supported (Evans 1963, Dietrich and Deitz 1993, Hamilton 1999, Cryan 2005). Synapomorphies of the Membracoidea include the enlarged, transverse metathoracic coxae and a pair of rod-shaped lateral apodemes associated with the scutellar suture (Dietrich and Deitz 1993).

The family Cicadellidae (leafhoppers), with over 21,000 described species placed in more than 120 family-group taxa (Oman et al. 1990, Hamilton 1999), includes many species of economic importance because they are vectors of pathogens of cultivated plants (Nielson 1985). According to the morphological phylogeny of Hamilton (1983) and the molecular phylogeny (28S rDNA) of Dietrich et al. (2001), Cicadellidae is a paraphyletic group because treehoppers (Aetalionidae and Membracidae) are derived from leafhoppers. Taxonomically, cicadellids can be distinguished from other membracoids by the mesanepisternum without a hooklike process, separated from the katepisternum by a suture, and hind tibia with setae of longitudinal rows usually large and conspicuous (Dietrich 2005). With over 2,000 known species and a cosmopolitan distribution, Cicadellinae (sharpshooters) is the third largest subfamily of the Cicadellidae (Mejdalani 1998, Takiya 2007, McKamey 2007). According to Young (1968, 1977, 1986), this subfamily is divided into two tribes, a cosmopolitan Cicadellini and a New World Proconiini. Sharpshooters feed on the low-nutrient xylem sap of vascular plants. Some species of this group are important vectors of xylem-borne phytopathogenic bacteria (Redak et al. 2004).

The genus *Subrasaca* Young, 1977 belongs to the Cicadellini. *Subrasaca* has records from Brazil and Argentina, as well as dubious records of *Subrasaca monacha* from Colombia (Young 1977, McKamey 2007, Silva et al. 2013b). Species records are mostly from the Atlantic Rainforest. *Subrasaca* comprises currently 14 species (Silva et al. 2013a,b): *Subrasaca atronasa* Young, 1977, *Subrasaca austera* Young, 1977, *Subrasaca bimaculata* Silva, Cavichioli & Mejdalani, 2013a, *Subrasaca constricta* Silva, Cavichioli & Mejdalani, 2013a, *Subrasaca curvovittata* (Stål, 1862), *Subrasaca diminuta* Silva, Cavichioli & Mejdalani, 2013b, *Subrasaca flavolineata* (Signoret, 1855), *Subrasaca flavoornata* (Stål, 1862), *Subrasaca ignicolor* (Signoret, 1854) (type species), *Subrasaca monacha* (Melichar, 1951), *Subrasaca nigriventris* (Signoret, 1855), *Subrasaca rachelae* Silva, Cavichioli & Mejdalani, 2013b, *Subrasaca rhienetta* (Signoret, 1854), and *Subrasaca rubra* Silva, Cavichioli & Mejdalani, 2013b.

Taxonomically, *Subrasaca* differs from other genera of the Cicadellini by the following combination of male genital characteristics (Silva et al. 2013b): (1) aedeagus usually short and dorsally expanded; (2) styles (parameres) with distinct preapical lobe; (3) paraphyses with two or four rami (except in *Subrasaca monacha*, with only one ramus); and (4) subgenital plates connected to each other at base by a triangular membranous area, not extending posteriorly as far as pygofer apex. *Subrasaca* species are generally quite colorful and range in length from 4.8 to 7.7 mm. Young (1977: 445), based on overall similarity, included *Subrasaca* in his *Juliaca* group of genera, which also includes *Juliaca* Melichar, 1926, *Mesogonia* Melichar, 1926, *Rotigonalia* Young, 1977, *Geitogonalia* Young, 1977, *Plerogonalia* Young, 1977, *Scopogonalia* Young, 1977, *Cyclogonia* Melichar, 1926, *Beirneola* Young, 1977, and *Fusigonalia* Young, 1977.

Here we use morphological data of the head, thorax, male and female genitalia to investigate the phylogenetic relationships among the species of *Subrasaca*. Among our outgroups, we included four genera of the *Juliaca* group (*Cyclogonia*, *Juliaca*, *Geitogonalia*, and *Scopogonalia*).

## Material and methods

### Specimens for the study

Specimens of 12 of the 14 described species of *Subrasaca* were studied (*Subrasaca atronasa* and *Subrasaca monacha* were not obtained and thus coded based on Young 1977 and Wilson et al. 2009). The matrix includes 20 terminal taxa (14 *Subrasaca* species and six outgroups). The outgroups are four representatives of the *Juliaca* generic group [*Cyclogonia caeliguttata* Mejdalani & Nessimian, 1991, *Juliaca* sp., *Geitogonalia quatuordecimmaculata* (Taschenberg, 1884), *Scopogonalia subolivacea* (Stål, 1862)], *Versigonalia ruficauda* (Walker, 1851), and a member of the Proconiini, *Tretogonia cribrata* Melichar, 1926, which was employed for rooting the trees.

The studied specimens belong to the following institutions: Departamento de Entomologia, Museu Nacional, Universidade Federal do Rio de Janeiro (MNRJ, Rio de Janeiro); Coleção Entomológica Prof. José Alfredo P. Dutra, Departamento de Zoologia, Instituto de Biologia, Universidade Federal do Rio de Janeiro (DZRJ, Rio de Janeiro); and Coleção de Entomologia Pe. Jesus S. Moure, Departamento de Zoologia, Setor de Ciências Biológicas, Universidade Federal do Paraná (DZUP, Curitiba). The number of specimens examined of each terminal taxon, their geographical distribution, and collections are listed in Table 1.

**Table 1.** Taxa included in the phylogenetic analysis of *Subrasaca* (in bold) and outgroups. The number of females and males examined, their distribution (Brazilian states), and collections are provided for each taxon.

Taxon	Females	Males	Distribution	Collection
*Cyclogonia caeliguttata* Mejdalani & Nessimian, 1991	2	2	RJ	MNRJ
*Juliaca* sp.	2	2	RJ	MNRJ
*Geitogonalia quatuordecimmaculata* (Taschenberg, 1884)	2	2	RJ	MNRJ
*Scopogonalia subolivacea* (Stål, 1862)	2	2	RJ, MG	MNRJ
*Versigonalia ruficauda* (Walker, 1851)	2	2	RJ	MNRJ
*Tretogonia cribrata* Melichar, 1926*	2	2	RJ	MNRJ
*Subrasaca atronasa* Young, 1977**	–	–	–	–
***Subrasaca austera* Young, 1977**	2	1	SC	DZUP
***Subrasaca bimaculata* Silva et al., 2013**	19	26	MG, SP, PR	DZRJ, DZUP, MNRJ
***Subrasaca constricta* Silva et al., 2013**	2	3	BA	DZUP, MNRJ
***Subrasaca curvovittata* (Stål, 1862)**	11	6	RJ	DZRJ, DZUP, MNRJ
***Subrasaca diminuta* Silva et al., 2013**	8	6	SP, PR	DZUP, MNRJ
***Subrasaca flavolineata* (Signoret, 1855)**	9	15	RJ	DZRJ, DZUP, MNRJ
***Subrasaca flavoornata* (Stål, 1862)**	6	2	RJ	MNRJ
***Subrasaca ignicolor* (Signoret, 1854)**	22	14	RJ, SP	MNRJ
*Subrasaca monacha* (Melichar, 1951)**	–	–	–	–
***Subrasaca nigriventris* (Signoret, 1855)**	10	10	RJ	MNRJ
***Subrasaca rachelae* Silva et al., 2013**	19	12	ES	DZRJ, DZUP, MNRJ
***Subrasaca rhienetta* (Signoret, 1854)**	3	3	RJ, SP	MNRJ
***Subrasaca rubra* Silva et al., 2013**	7	10	MG, RJ, SP	DZRJ, DZUP, MNRJ

Brazilian states: BA – Bahia; ES – Espírito Santo; MG – Minas Gerais; PR – Paraná; RJ – Rio de Janeiro; SC – Santa Catarina; SP – São Paulo. DZRJ – Departamento de Zoologia, Universidade Federal do Rio de Janeiro; DZUP – Departamento de Zoologia, Universidade Federal do Paraná; MNRJ – Museu Nacional, Universidade Federal do Rio de Janeiro; * root of the phylogenetic analysis; ** coded based on Young (1977) and Wilson et al. (2009).

### Techniques for preparation of specimens and terminology

The techniques for preparation of male and female genital structures follow Oman (1949) and Mejdalani (1998), respectively. The dissected parts are stored in small vials with glycerin, as suggested by Young and Beirne (1958). The first and second pair of valvulae of the ovipositor were mounted on temporary slides with glycerin. The descriptive terminology adopted herein follows mainly Young (1977), except for the facial areas of the head (Hamilton 1981, Mejdalani 1993, 1998) and the female genitalia (Nielson 1965, Hill 1970).

### Cladistic analysis

Morphological characters of the head, thorax, male and female genitalia were included in the unpolarized matrix (Nixon and Carpenter 1993), which was assembled using the Nexus Data Editor (Page 2001). Hypotheses of primary homology were proposed based on the topological identity of the structures (Pinna 1991). All characters were initially scored equal weights. Character states were scored as underscores (_) when inapplicable or as question marks (?) when unavailable. The *heuristic search algorithm*, as implemented in PAUP* 4.0 (Swofford 2002), was employed for searching the most parsimonious trees. The successive weighting procedure (Carpenter 1988, 1994) was based on the maximum rescaled consistency index (rc) of the characters (Farris 1969, 1989). The strict consensus method was employed for all original most parsimonious trees. Clade support was estimated by computing 10.000 bootstrap replicates (Felsenstein 1985) with heuristic search in PAUP* 4.0 and by decay indices (Bremer 1988, 1994) in TreeRot 3.0 (Sorenson and Franzosa 2007). Autapomorphic characters were included in the matrix, as suggested by Yeates (1992), but we provide consistency index (CI) values considering all characters as well as only the informative ones.

## Results and discussion

**Figure 1.**
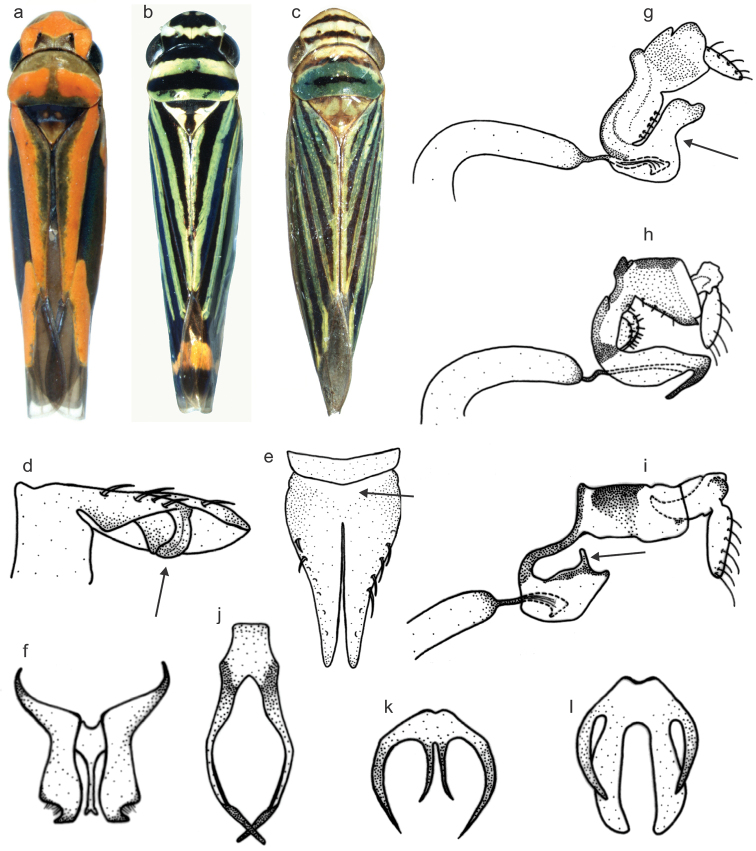
Examples of characters for the phylogenetic analysis of *Subrasaca* (external morphology and male genitalia). **a** body of *Subrasaca rachelae* (length 5.3 mm): rounded anterior margin of crown (character 1, state 0), maculae on lateroapical portions of crown (c6, s1), pair of moderately oblique maculae on pronotum (c21, s2) **b** *Subrasaca flavolineata* (length 5.4 mm): mesonotum with T-shaped macula (c22, s2), longitudinal stripes on forewings (c25, s1) **c** *Subrasaca constricta* (length 5.7 mm): pronounced anterior margin of crown (c1, s1) **d** pygofer lobe of *Subrasaca constricta*, dorsal view: dorsoapical process (c36, s1; arrowed) **e** subgenital plates of *Subrasaca bimaculata*: membranous basal area (c38, s1; arrowed) **f** *Subrasaca nigriventris*: styles with preapical lobe (c40, s1) and apex transversely truncate (c42, s0), stalk of connective clearly differentiated, not extending beyond apex of styles (c44, s1) **g** aedeagus of *Subrasaca constricta*: dorsal lobe (c46, s1) with constriction (c47, s1; arrowed) **h** aedeagus of *Subrasaca nigriventris*: shaft longer than high (c48, s1), pair of spiniform apical processes (c51, s2) **i** aedeagus of *Subrasaca rachelae*: pair of preapical processes (c52, s1; arrowed) **j** *Subrasaca rubra*: paraphyses with two rami (c55, s1) **k** *Subrasaca curvovittata*: paraphyses with four rami (c55, s2), inner rami small and narrow (c56, s0) **l** *Subrasaca bimaculata*: inner rami of paraphyses broader and larger than outer rami (c56, s1).

The data matrix (Table 2) consists of 72 morphological characters, 35 of the external morphology, 25 of the male genitalia, and 12 of the female genitalia. Among these characters, 51 are binary and 21 are multistate, being 52 informative for the parsimony analysis. The characters, their states, and *ci* greater than 0.5 are listed below. Although many of the characters are based on color patterns, these are consistent intraspecifically in *Subrasaca*. Figures 1 (external morphology and male genitalia) and 2 (female genitalia) provide some examples of characters employed in the phylogenetic analysis.

**Figure 2.**
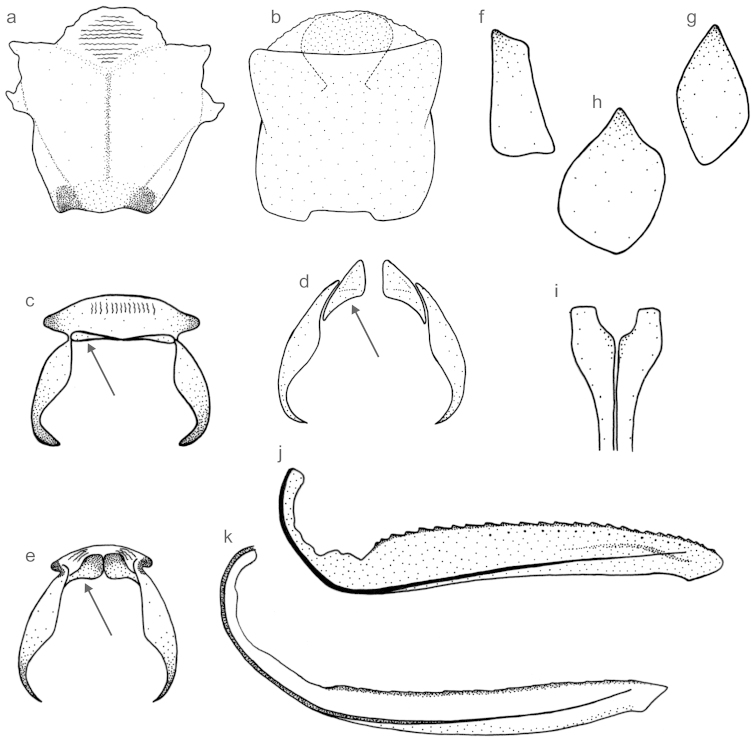
Examples of characters for the phylogenetic analysis of *Subrasaca* (female genitalia). **a** sternite VII of *Subrasaca bimaculata*: convex posterior margin at median portion (character 61, state 1) **b** sternite VII of *Subrasaca rubra*: concave posterior margin at median portion (c61, s0) **c** *Subrasaca rachelae*: smooth sclerites of “inner” sternite VIII (c65, s0) with linear aspect (c66, s2; arrowed) **d** *Subrasaca flavoornata*: sclerites of “inner” sternite VIII with oblique aspect (c66, s4; arrowed) **e** *Subrasaca flavolineata*: punctuated sclerites of “inner” sternite VIII (c65, s1; arrowed) **f** *Juliaca* sp.: valvifer I subrectangular (c68, s2) **g** *Subrasaca diminuta*: valvifer I ellipsoid (c68, s4) **h** *Subrasaca curvovittata*: valvifer I gutiform (c68, s6) **i** *Subrasaca rhienetta*: valvulae I with expanded base (c69, s0) **j** *Subrasaca constricta*: valvula II with obtuse apex (c70, s0) and convex dorsal margin (c72, s0) **k** *Subrasaca rubra*: valvula II with acute apex (c70, s1), linear and indistinct teeth (c71, s1), and rectilinear dorsal margin (c72, s1).

**Table 2.** Data matrix for the phylogenetic analysis of *Subrasaca* (in bold) and outgroup taxa. (_) codes for inapplicable states, (?) for unavailable data, (A) for state 10, (B) for 11, and (C) for 12. Outgroup genera are *Cyclogonia*, *Juliaca*, *Geitogonalia*, *Scopogonalia*, *Versigonalia*, and *Tretogonia* (root).

Taxa	Characters
	1 2 3 4 5 6 7
	1 2 3 4 5 6 7 8 9 0 1 2 3 4 5 6 7 8 9 0 1 2 3 4 5 6 7 8 9 0 1 2 3 4 5 6 7 8 9 0 1 2 3 4 5 6 7 8 9 0 1 2 3 4 5 6 7 8 9 0 1 2 3 4 5 6 7 8 9 0 1 2
*Cyclogonia caeliguttata*	0 2 1 0 _ 0 0 1 2 0 1 0 0 0 _ 0 _ 0 1 2 0 5 0 3 0 _ _ 0 _ _ _ 0 _ 1 0 0 0 0 0 1 0 1 1 2 1 0 _ 1 0 _ _ 0 0 1 1 _ 0 0 _ 0 1 0 0 _ _ _ 0 4 1 1 0 0
*Juliaca* sp.	1 1 1 0 _ 0 0 0 _ _ _ 1 1 0 _ 0 _ 1 0 _ _ 6 0 1 0 _ _ 0 _ _ _ 0 _ 1 0 0 0 0 1 0 _ 1 1 1 1 0 _ 1 0 _ _ 0 0 1 1 _ 0 0 _ 0 3 0 0 _ _ _ 0 2 1 1 0 1
*Geitogonalia quatuordecimmaculata*	0 1 4 1 2 0 0 1 0 0 0 0 0 0 _ 0 _ 0 1 0 1 0 0 2 0 _ _ 0 _ _ _ 0 _ 1 1 0 0 0 0 1 1 1 0 2 1 0 _ 1 1 1 0 0 0 1 1 _ 0 0 _ 0 2 0 0 _ _ _ 0 3 0 1 0 0
*Scopogonalia subolivacea*	1 2 C 0 _ 0 0 0 _ _ _ 0 0 0 _ 0 _ 0 0 _ _ 9 0 1 0 _ _ 0 _ _ _ 0 _ 1 0 0 1 1 0 0 _ 0 1 _ _ 0 _ 1 1 1 2 0 0 1 1 _ 0 0 _ 0 0 0 1 0 1 2 0 4 0 1 0 0
*Versigonalia ruficauda*	0 2 B 0 _ 1 0 0 _ _ _ 0 1 0 _ 0 _ 0 0 _ _ 8 0 4 0 _ _ 1 0 2 0 0 _ 1 2 0 1 0 0 0 _ 1 1 1 1 0 _ 1 0 _ _ 0 1 0 _ _ _ _ _ _ 0 0 0 _ _ _ 0 4 0 1 1 2
*Tretogonia cribrata* (root)	0 2 2 0 _ 0 0 0 _ _ _ 0 0 0 _ 0 _ 0 0 _ _ 5 0 3 0 _ _ 0 _ _ _ 0 _ 0 1 0 1 0 1 1 0 3 1 1 1 0 _ 1 0 _ _ 0 1 0 _ _ _ _ _ _ 0 0 1 0 0 5 1 0 0 0 0 0
***Subrasaca ignicolor***	0 0 6 0 _ 0 0 1 0 1 0 0 0 0 _ 0 _ 0 1 0 0 0 0 2 0 _ _ 0 _ _ _ 0 _ 1 0 0 0 1 0 1 1 2 1 1 1 1 0 0 0 _ _ 0 0 1 1 _ 0 0 _ 0 0 0 1 1 1 2 0 4 0 0 0 0
***Subrasaca rhienetta***	1 2 3 1 0 0 1 0 _ _ _ 1 1 0 _ 0 _ 1 0 _ _ 0 0 0 0 _ _ 0 _ _ _ 1 0 1 0 0 0 1 0 1 0 2 1 1 1 1 1 0 0 _ _ 0 0 1 1 _ 0 0 _ 0 0 1 1 1 0 4 0 4 0 1 0 1
***Subrasaca nigriventris***	0 0 1 0 _ 1 0 1 0 1 1 0 0 0 _ 0 _ 0 1 0 2 0 0 0 0 _ _ 0 _ _ _ 0 _ 1 0 0 0 1 0 1 0 0 1 1 1 0 _ 1 1 1 2 0 0 1 1 _ 0 0 _ 0 0 1 1 1 0 0 0 4 0 0 0 0
***Subrasaca flavolineata***	1 1 8 1 1 0 1 0 _ _ _ 1 1 0 _ 1 1 0 0 _ _ 2 0 1 1 0 0 1 0 0 0 0 _ 1 0 0 0 1 0 1 1 1 1 1 1 1 0 0 0 _ _ 0 0 1 1 _ 0 1 1 0 0 1 1 1 1 1 0 4 1 0 0 0
	1
***Subrasaca monacha***	0 0 2 0 _ 0 0 1 1 1 0 0 0 0 _ 0 _ 0 0 _ _ 5 0 3 0 _ _ 0 _ _ _ 0 _ 1 ? 0 0 ? 0 1 0 2 1 1 1 0 _ 1 1 0 3 0 0 1 0 _ 0 0 _ 0 ? ? ? ? ? ? ? ? ? ? ? ?
***Subrasaca flavoornata***	0 0 9 0 _ 0 0 1 0 1 0 0 0 0 _ 0 _ 0 1 0 3 0 0 0 0 _ _ 0 _ _ _ 0 _ 1 0 0 0 1 0 1 0 1 1 0 1 0 _ 1 1 1 1 0 0 1 1 _ 0 0 1 1 1 0 1 1 1 4 1 4 0 1 0 0
	3 1
***Subrasaca curvovittata***	1 1 7 1 0 0 1 0 _ _ _ 1 1 0 _ 1 1 0 0 _ _ 1 0 0 1 0 0 1 1 0 0 0 _ 1 0 0 0 1 0 1 0 1 1 1 1 1 0 0 0 _ _ 0 0 1 2 0 0 0 _ 0 0 1 1 1 1 4 1 6 1 0 0 0
	1
***Subrasaca atronasa***	1 0 ? 0 _ 0 0 1 2 1 0 0 0 1 0 0 _ 0 0 _ _ 5 1 1 0 _ _ 0 _ _ _ 1 1 1 0 0 0 1 0 1 0 2 1 1 1 1 0 0 0 _ _ 0 0 1 1 _ 0 0 _ 0 ? ? ? ? ? ? ? ? ? ? ? ?
***Subrasaca austere***	1 0 A 1 3 0 0 1 2 1 0 0 0 1 1 0 _ 0 0 _ _ 5 1 3 0 _ _ 0 _ _ _ 0 _ 1 0 0 0 1 0 1 0 2 1 1 1 1 0 0 0 _ _ 0 0 1 1 _ 0 0 _ 0 0 0 1 1 0 0 0 4 0 1 0 1
	1
***Subrasaca constricta***	1 0 1 0 _ 0 1 0 _ _ _ 1 1 0 _ 1 0 0 0 _ _ 7 0 1 1 2 1 1 0 0 1 0 _ 1 0 1 0 1 0 1 0 0 1 1 0 1 1 0 0 _ _ 0 0 1 1 _ 0 1 0 0 0 0 1 1 1 0 0 5 0 0 0 0
***Subrasaca bimaculata***	1 1 6 1 0 0 1 0 _ _ _ 1 1 0 _ 1 1 0 0 _ _ 4 0 1 1 1 0 1 0 0 0 0 _ 1 0 0 0 1 0 1 0 0 1 1 1 1 0 0 0 _ _ 0 0 1 2 1 0 0 _ 0 1 0 0 _ _ _ 0 1 1 0 0 0
	1 1 2
***Subrasaca rachelae***	0 0 1 0 _ 1 0 1 0 1 1 0 0 0 _ 0 _ 0 1 0 2 3 0 3 0 _ _ 0 _ _ _ 0 _ 1 0 0 0 1 0 1 0 0 1 1 0 1 0 0 0 _ _ 1 0 1 1 _ 0 0 _ 1 1 1 1 1 0 2 0 4 0 1 0 0
***Subrasaca diminuta***	0 0 5 0 _ 0 0 1 0 1 0 0 0 0 _ 0 _ 0 1 0 0 0 0 0 0 _ _ 0 _ _ _ 0 _ 1 0 0 0 1 0 1 0 2 1 1 1 1 0 0 0 _ _ 0 0 1 1 _ 1 0 _ 0 0 0 1 1 0 2 0 4 0 0 0 0
***Subrasaca rubra***	0 0 0 0 _ 0 0 0 _ _ _ 0 0 0 _ 0 _ 0 1 0 3 0 0 2 0 _ _ 0 _ _ _ 0 _ 1 1 0 0 1 0 1 0 0 1 1 1 1 0 0 0 _ _ 0 0 1 1 _ 0 0 _ 0 0 0 1 1 1 3 1 4 0 1 1 1

### Morphological characters of the phylogenetic analysis

#### External morphology and coloration

**1.Shape of anterior margin of crown, dorsal view:** (0) rounded (Fig. 1a), (1) pronounced with a triangular shape (Fig. 1c).

**2.Position of ocelli on crown:** (0) slightly anterior to imaginary line between anterior eye angles (Fig. 1c); (1) posterior to imaginary line between eye angles; (2) on imaginary line between eye angles.

**3.Color of face:** (0) black; (1) yellow; (2) light brown; (3) black with yellow central region enclosing black macula; (4) black with cream central macula and two orange maculae on anterior portion; (5) black with orange macula on posterior portion; (6) black with orange macula on anterior portion; (7) yellow with black Y-shaped macula; (8) yellow with black central portion enclosing yellow macula; (9) cream with black central portion and anterior region with orange macula; (A) yellow with brown streaks and median stripe; (B) black with orange lateral portions; (C) yellow with small black maculae on anterior portion. ci = 0.9.

**4.Macula or maculae, originated from face, limited to central portion of apex of crown:** (0) absent; (1) present (Fig. 1b).

**5.Color of macula or maculae, originated from face, limited to central portion of apex of crown:** (0) yellow; (1) brown with yellow (Fig. 1b); (2) orange; (3) brown. ci = 1.

**6.Maculae on lateroapical portions of crown, originated from face:** (0) absent; (1) present (Fig. 1a). ci = 0.5.

**7.Dark brown to black transversal band on anterior portion of crown:** (0) absent; (1) present (Fig. 1b). ci = 1.0.

**8.Transverse band on middle portion of crown:** (0) absent; (1) present. ci = 0.5.

**9.Color of transverse band on middle portion of crown:** (0) orange; (1) light brown; (2) whitish-yellow; (3) yellow. ci = 0.6.

**10.Aspect of transverse band on middle portion of crown:** (0) incomplete; (1) complete. ci = 1.0.

**11.Lateral portions of median transverse band reaching posterior margin of crown:** (0) absent; (1) present. ci = 0.5.

**12.Dark brown to black transverse band on posterior portion of crown:** (0) absent; (1) present (Fig. 1b). ci = 0.5.

**13.Dark brown to black transverse band on anterior portion of pronotum:** (0) absent; (1) present (Fig. 1b).

**14.Whitish-yellow transverse band on middle portion of pronotum:** (0) absent; (1) present. ci = 1.

**15.Band on middle portion of pronotum with strong concavity:** (0) absent; (1) present. ci = 1.

**16.Dark brown to black transverse band located before base of pronotum (posterior margin):** (0) absent; (1) present (Fig. 1b). ci = 1.

**17.Aspect of dark brown to black transverse band located before base of pronotum (posterior margin):** (0) narrow (Fig. 1c); (1) thick (Fig. 1b). ci = 1.

**18.Dark brown to black transverse band at base of pronotum (posterior margin):** (0) absent; (1) present. ci = 0.5.

**19.Pair of maculae on central portion of pronotum:** (0) absent; (1) present.

**20.Color of pair of maculae on central portion of pronotum:** (0) orange; (1) yellow; (2) blue. ci = 1.

**Figure 3.**
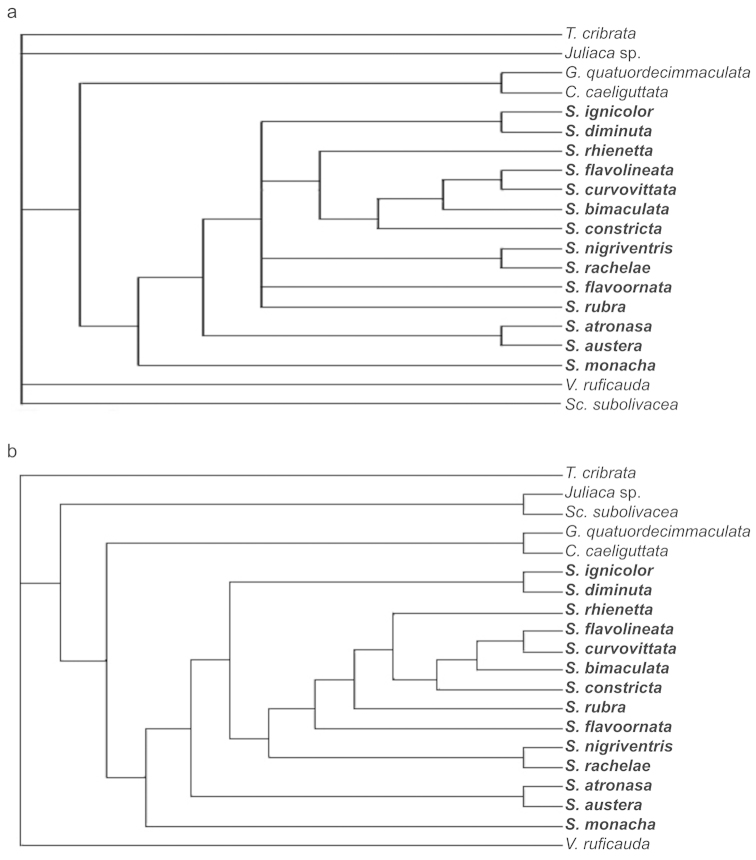
**a** Strict consensus of the six equally most parsimonious trees of the phylogenetic analysis of *Subrasaca* and outgroup taxa **b** Most parsimonious tree obtained with the successive weighting procedure; length = 80, consistency index = 0.8249 (excluding uninformative characters = 0.7199), retention index = 0.7641, rescaled consistency index = 0.6303. Outgroup genera are *Cyclogonia*, *Geitogonalia*, *Juliaca*, *Scopogonalia*, *Versigonalia*, and *Tretogonia* (root).

**21.Position of pair of yellow or orange maculae on pronotum:** (0) strongly oblique and restricted to sides of pronotum; (1) strongly oblique and reaching central portion of pronotum; (2) moderately oblique (Fig. 1a); (3) not oblique. ci = 1.

**Figure 4.**
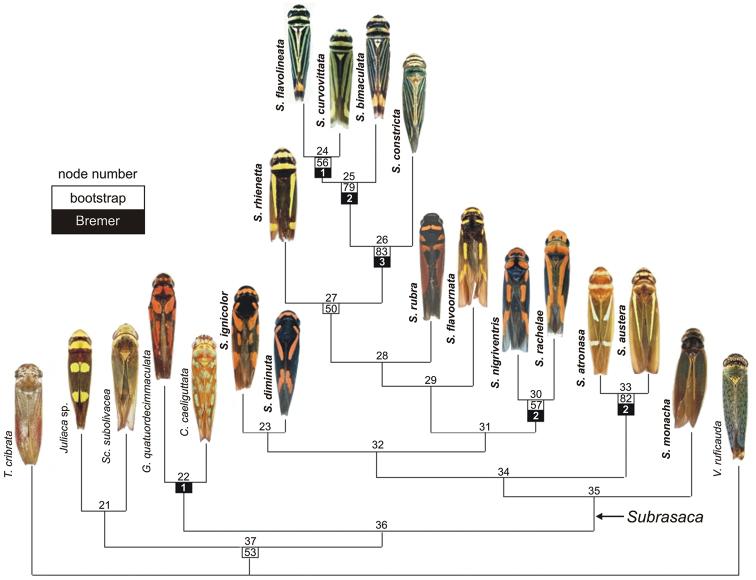
One of the most parsimonious trees of the phylogenetic analysis of *Subrasaca* and outgroup taxa; this is also the single tree obtained with the successive weighting procedure. Length = 197, consistency index = 0.6091 (excluding uninformative characters = 0.5389), retention index = 0.5722, rescaled consistency index = 0.3486. Species of *Subrasaca* in bold. Apomorphies are given in Table 3. Most sharpshooter images from Wilson et al. (2009).

**22.Color of mesonotum:** (0) entirely black; (1) black with yellow scutellum; (2) yellow with black T-shaped macula (Fig. 1b); (3) brown with brownish-yellow scutellum and two anterior maculae (Fig. 1a); (4) black with yellow scutellum and two anterior maculae; (5) light brown; (6) dark brown with yellow apex; (7) yellow (Fig. 1c); (8) black with irregular yellow maculae; (9) yellow with black maculae on lateroanterior portions. ci = 0.9.

**23.Texture of forewings:** (0) coriaceous (Fig. 1a–c); (1) translucent. ci = 1.

**24.Color of basal portion of clavus, reaching apex of scutellum:** (0) black; (1) yellow (Fig. 1b, c); (2) orange; (3) light brown (Fig. 1a); (4) blue.

**25.Set of dark brown to black longitudinal stripes on forewings:** (0) absent; (1) present (Fig. 1b, c). ci = 1.

**26.Number of dark brown to black longitudinal stripes on forewings:** (0) four; (1) six; (2) eight. ci = 1.

**27.Position of longitudinal stripe on forewings:** (0) not along outer edge of inner apical cell; (1) along outer edge of inner apical cell. ci = 1.

**28.Transverse band on anteapical portion of forewings:** (0) absent; (1) present. ci = 0.5.

**29.Aspect of transverse band on anteapical portion of forewings:** (0) not connected to yellow longitudinal stripe; (1) connected to yellow longitudinal stripe. ci = 1.

**30.Color of transverse band on anteapical portion of forewings:** (0) yellow; (1) orange; (2) brown. ci = 1.

**31.Extension of transverse band on anteapical portion of forewings:** (0) reaching the four anteapical cells; (1) 1/2 of the width of wing, reaching a maximum of two anteapical cells. ci = 1.

**32.Transverse band of corium on region of apex of clavus:** (0) absent; (1) present. ci = 0.5.

**33.Color of transverse band of corium on apex of clavus:** (0) yellow; (1) whitish-yellow. ci = 1.

**34.Extension of hind legs at rest position:** (0) not reaching posterior margin of lateral lobe of pronotum; (1) reaching posterior margin of lateral lobe of pronotum. ci = 1.

**35.Color of legs:** (0) yellow; (1) brown; (2) red. ci = 0.5.

**Male genitalia**

**36.Dorsoapical process of pygofer:** (0) absent; (1) present (Fig. 1d). ci = 1.

**37.Ventroapical process of pygofer:** (0) absent; (1) present. ci = 0.5.

**38.Triangular membranous area uniting subgenital plates basally:** (0) absent; (1) present (Fig. 1e). ci = 0.5.

**39.Extension of subgenital plates in relation to pygofer:** (0) not extending beyond apex of pygofer; (1) extending beyond apex of pygofer. ci = 0.5.

**40.Preapical lobe of styles:** (0) absent; (1) present (Fig. 1f). ci = 0.5.

**41.Styles, length of portion posterior to preapical lobe:** (0) less than 1/3 of style length (Fig. 1f); (1) 1/3 of style length.

**42.Shape of apex of styles, dorsal view:** (0) transversely truncated (Fig. 1f); (1) obliquely truncated; (2) obtuse; (3) forked.

**43.Shape of connective:** (0) T-shaped; (1) Y-shaped (Fig. 1f). ci = 1.

**44.Aspect of stalk of connective:** (0) very short, not clearly differentiated; (1) clearly differentiated, not extending beyond apex of styles (Fig. 1f); (2) clearly differentiated, extending beyond apex of styles. ci = 1.

**45.Width of stalk of connective:** (0) similar to width of base of arms; (1) narrower than base of arms (Fig. 1f). ci = 0.5.

**46.Dorsal lobe of aedeagus:** (0) absent; (1) present (Fig. 1g).

**47.Constriction of dorsal lobe of aedeagus:** (0) absent; (1) present (Fig. 1g). ci = 0.5.

**48.Length of aedeagus:** (0) as long as high; (1) longer than high (Fig. 1h).

**49.Apical processes of aedeagus:** (0) absent; (1) present (Fig. 1h).

**50.Number of apical processes of aedeagus:** (0) one; (1) two. ci = 1.

**51.Shape of apical processes of aedeagus:** (0) pair of digitiform processes directed basally; (1) pair of small lobular processes; (2) pair of spiniform processes directed ventrally (Fig. 1h); (3) triangular projection directed ventrally. ci = 1.

**52.Pair of dorsally directed digitifom processes on preapical portion of aedeagus:** (0) absent; (1) present (Fig. 1i). ci = 1.

**53.Pair of basal processes of aedeagus:** (0) absent; (1) present. ci = 1.

**54.Paraphyses:** (0) absent; (1) present (Fig. 1j–l). ci = 1.

**55.Number of paraphyses rami:** (0) one; (1) two (Fig. 1j); (2) four (Fig. 1k, l). ci = 0.6.

**56.Aspect of inner rami of paraphyses with four rami:** (0) narrower and smaller than outer rami (Fig. 1k); (1) broader and larger than outer rami (Fig. 1l); (2) width and length similar to outer rami. ci = 1.

**57.Spiniform process of paraphyses:** (0) absent; (1) present. ci = 1.

**58.Process on median portion of rami of paraphyses:** (0) absent; (1) present. ci = 0.5.

**59.Number of processes on median portion of paraphyses rami:** (0) one on each ramus; (1) two on each ramus. ci = 1.

**60.Apical processes of paraphyses:** (0) absent; (1) present. ci = 0.5.

**Female genitalia**

**61.Aspect of median portion of posterior margin of sternite VII:** (0) concave (Fig. 2b); (1) convex (Fig. 2a); (2) concave with dentiform projection; (3) convex with short triangular projection. ci = 0.5.

**62.Distinctly sclerotized area on each side of anterior margin of sternite VII:** (0) absent; (1) present.

**63.Sclerites of “inner” sternite VIII:** (0) absent; (1) present (Fig. 2c–e).

**64.Number of sclerites of “inner” sternite VIII:** (0) one; (1) two (Fig. 2c–e). ci = 1.

**65.Texture of sclerites of “inner” sternite VIII:** (0) smooth (Fig. 2c); (1) punctuated (Fig. 2d, e).

**66.Shape of sclerites of “inner” sternite VIII:** (0) triangular; (1) somewhat quadrangular (Fig. 2e); (2) linear (Fig. 2c); (3) coniform; (4) oblique (Fig. 2d); (5) broad, narrowed posteriorly, and with apex located between bases of ovipositor valvulae I. ci = 0.7.

**67.Sclerites of “inner” sternite VIII directed ventrally:** (0) absent; (1) present.

**68.Shape of valvifers I, lateral view:** (0) quadrangular; (1) trapezoidal; (2) subrectangular (Fig. 2f); (3) gutiform with lobe on distal half of dorsal margin; (4) elliptical (Fig. 2g); (5) subtriangular; (6) gutiform, posteriorly expanded (Fig. 2h). ci = 1.

**69.Aspect, in ventral view, of basal portion of valvulae I of ovipositor:** (0) expanded (Fig. 2i); (1) continuous, without expansion.

**70.Aspect of apex of valvulae II of ovipositor:** (0) obtuse (Fig. 2j); (1) acute (Fig. 2k).

**71.Shape of teeth of valvulae II of ovipositor:** (0) triangular and distinct (Fig. 2j); (1) linear and indistinct (Fig. 2k). ci = 0.5.

**72.Aspect of dorsal margin of valvulae II of ovipositor:** (0) convex (Fig. 2j); (1) rectilinear (Fig. 2k); (2) concave (Young 1977: Fig. 881k).

### Main aspects and discussion of the phylogenetic analysis

The analysis with equal weights resulted in six most parsimonious trees with length = 197, consistency index (CI) = 0.6091 (excluding uninformative characters = 0.5389), retention index (RI) = 0.5722, and rescaled consistency index (RC) = 0.3486. The trees differ from one another (1) in the position of *Versigonalia ruficauda* (outgroup), (2) positions of *Subrasaca rubra* and *Subrasaca flavoornata*, which appear as sister groups or not, and (3) positions of *Subrasaca rubra*, *Subrasaca flavoornata*, *Subrasaca nigriventris*, and *Subrasaca rachelae*. These four species formed a clade with *Subrasaca ignicolor* + *Subrasaca diminuta* in two trees. A strict consensus of the six trees is given in Fig. 3a.

The successive weighting analysis yielded one tree, which is also one of the six original trees, with length = 80, CI = 0.8249 (excluding uninformative characters = 0.7199), RI = 0.7641, and RC = 0.6303 (Figs 3b, 4). Thirty-two characters had maximum weight (= 1.0) and 40 had lower weights. Twenty characters were parsimony-uninformative. Figure 4 gives bootstrap estimates (when > 50%) and Bremer support indices for the clades recovered under equal weights. Apomorphies of this tree are given in Table 3.

**Table 3.** Apomorphy list for clades of Fig. 4 of the phylogenetic analysis of *Subrasaca* and outgroup taxa. Non-homoplastic characters are in bold.

Node or terminal taxon	Apomorphies
37	37(0), **53(0)**, **54(1)**
21	1(1), 22(6), 24(1), 65(1)
36	8(1), 40(1), **64(1)**
22	**5(2)**, 19(1), **44(2)**, **51(0)**, 61(1)
35 (*Subrasaca*)	2(0), **10(1)**, 38(1), 42(2), 63(1)
34	46(1), 48(0)
33	1(1), 3(A), **5(3)**, 9(2), **14(1)**, **23(1)**, **33(1)**, 66(0), 72(1)
32	19(1), 22(0), 24(0)
23	3(5), 70(0)
31	**21(2)**, 42(0)
30	6(1), 11(1), 62(1)
29	**21(3)**, **51(1)**, 65(1), 66(4), 67(1)
28	8(0), 72(1)
27	1(1), 4(1), **7(1)**, 12(1), 13(1), 19(0), 47(1), 67(0)
26	**16(1)**, 22(1), 24(1), **25(1)**, 28(1), 70(0), 72(0)
25	2(1), 3(8), **17(1)**, 47(0), 55(2), **59(1)**, 69(1)
24	42(1), 62(1)
*Versigonalia ruficauda*	3(B), 6(1), 13(1), 22(8), 24(4), 28(1), **30(2)**, 35(2), 71(1), 72(2)
*Juliaca* sp.	2(1), 12(1), 13(1), 18(1), 39(1), 61(3), **68(2)**, 69(1), 72(1)
*Scopogonalia subolivacea*	3(C), 22(9), 37(1), 38(1), 42(0), 49(1), 63(1)
*Geitogonalia quatuordecimmaculata*	2(1), 3(4), 4(1), **21(1)**, 22(0), 24(2), 35(1), 41(1), **43(0)**, 49(1), 61(2), **68(3)**
*Cyclogonia caeliguttata*	9(2), 11(1), **20(2)**, 69(1)
*Subrasaca monacha*	3(2), 9(1), 49(1), **50(0)**, **51(3)**, 55(0)
*Subrasaca atronasa*	24(1), 32(1)
*Subrasaca austera*	4(1), **15(1)**
*Subrasaca ignicolor*	3(6), 24(2), 41(1), 65(1)
*Subrasaca diminuta*	**57(1)**
*Subrasaca nigriventris*	46(0), 48(1), 49(1), 66(0), 70(0)
*Subrasaca rachelae*	22(3), 24(3), 45(0), **52(1)**, 60(1), 61(1)
*Subrasaca flavoornata*	3(9), 42(1), **44(0)**, 46(0), 48(1), 49(1), 60(1), 61(1)
*Subrasaca rubra*	3(0), 24(2), 35(1), 66(3), 71(1)
*Subrasaca rhienetta*	2(2), 3(3), 18(1), 32(1), 42(2), 62(1), 65(0)
*Subrasaca constricta*	4(0), 22(7), **26(2)**, **27(1)**, **31(1)**, **36(1)**, 45(0), 58(1), 66(0), **68(5)**
*Subrasaca bimaculata*	22(4), **26(1)**, **56(1,2)**, 61(1), 63(0), **68(1)**
*Subrasaca flavolineata*	**5(1)**, 22(2), 41(1), 55(1), 58(1), 66(1)
*Subrasaca curvovittata*	3(7), **29(1)**, 67(1), **68(6)**

The monophyly of *Subrasaca* was recovered in all most parsimonious trees (Fig. 3a). This clade, however, is not robust (bootstrap < 50%, Bremer = 0) (Fig. 4). Phylogenetically, *Subrasaca* can be tentatively defined by the following synapomorphic traits of its groundplan: (1) ocelli located slightly anterad of the imaginary line between the anterior angles of eyes (character 2, state 0; Fig. 1c), (2) complete transverse band on middle portion of crown (character 10, state 1), (3) triangular membranous area uniting subgenital plates basally (character 38, state 1; Fig. 1e), (4) obtuse shape of apex of styles in dorsal view (character 42, state 2), and (5) sclerites of female “inner” sternite VIII present (character 63, state 1; Fig. 2c–e).

Other phylogenetic (e.g., Felix and Mejdalani 2011) or purely taxonomic (e.g., Mejdalani et al. 2014) studies on the Cicadellini highlighted the need for more precise definitions of various genera of this tribe. In the introduction of his impressive monograph on the New World Cicadellini, Young (1977: 10) expressed his perception of this problem as follows: “The Cicadellini are an intricate group. Their morphology suggests rapid radiation and often shows small discontinuities compared with those found in many of the Proconiini.” Small discontinuities are precisely what we have observed between *Subrasaca* and the genera here employed as outgroups. In any case, the cladistic analysis allowed us to propose a more objective definition of the genus.

Two clades appeared in all six most parsimonious trees and were fairly robust in the analysis (Fig. 4). The clade formed by the species with longitudinal dark brown to black stripes on the forewings (*Subrasaca bimaculata*, *Subrasaca constricta*, *Subrasaca curvovittata*, and *Subrasaca flavolineata*) had the highest percentage of bootstrap (= 83%) and was supported by seven apomorphic conditions (Table 3, node 26), including the conspicuous set of dark brown to black longitudinal stripes on the forewings (character 25, state 1; Fig. 1b, c). The Bremer support of this clade was 3. It shows the following internal relationships in all trees (Fig. 3a): (*Subrasaca constricta* (*Subrasaca bimaculata* (*Subrasaca flavolineata*, *Subrasaca curvovittata*))). These four species were described in detail by Silva et al. (2013a). The group is distributed in the Atlantic Forest from northeastern (state of Bahia) to southern Brazil (state of Paraná). The second clade, formed by *Subrasaca atronasa* + *Subrasaca austera*, is supported by nine apomorphic conditions (Table 3, node 33), including a whitish-yellow transverse band on middle portion of pronotum (character 14, state 1; Fig. 4, node 33). This clade also had relatively high bootstrap (= 82%) and Bremer (= 2) scores. These two species, which are known only from the state of Santa Catarina (Zanol and de Menezes 1982) in southern Brazil (Atlantic Forest), were described by Young (1977), who considered them “very close” to each other (Young 1977: 479).

Although with low support scores, the clades formed by *Subrasaca ignicolor* + *Subrasaca diminuta* and *Subrasaca nigriventris* + *Subrasaca rachelae* were recovered in all most parsimonious trees (Fig. 4, nodes 23 and 30, respectively). Unlike the species with longitudinal dark brown to black stripes on the forewings (node 26), those with contrasting orange marks (*Subrasaca diminuta* + *Subrasaca ignicolor*, *Subrasaca nigriventris* + *Subrasaca rachelae*, and *Subrasaca rubra*) did not form a monophyletic group in any of the six most parsimonious trees.
